# Supplementary figures and images for: Contrast normalization affects response time-course of visual interneurons
Source: PLoS One. 2023 Jun 9;18(6):e0285686. doi: 10.1371/journal.pone.0285686 (PMC10256145; doi:10.1371/journal.pone.0285686)

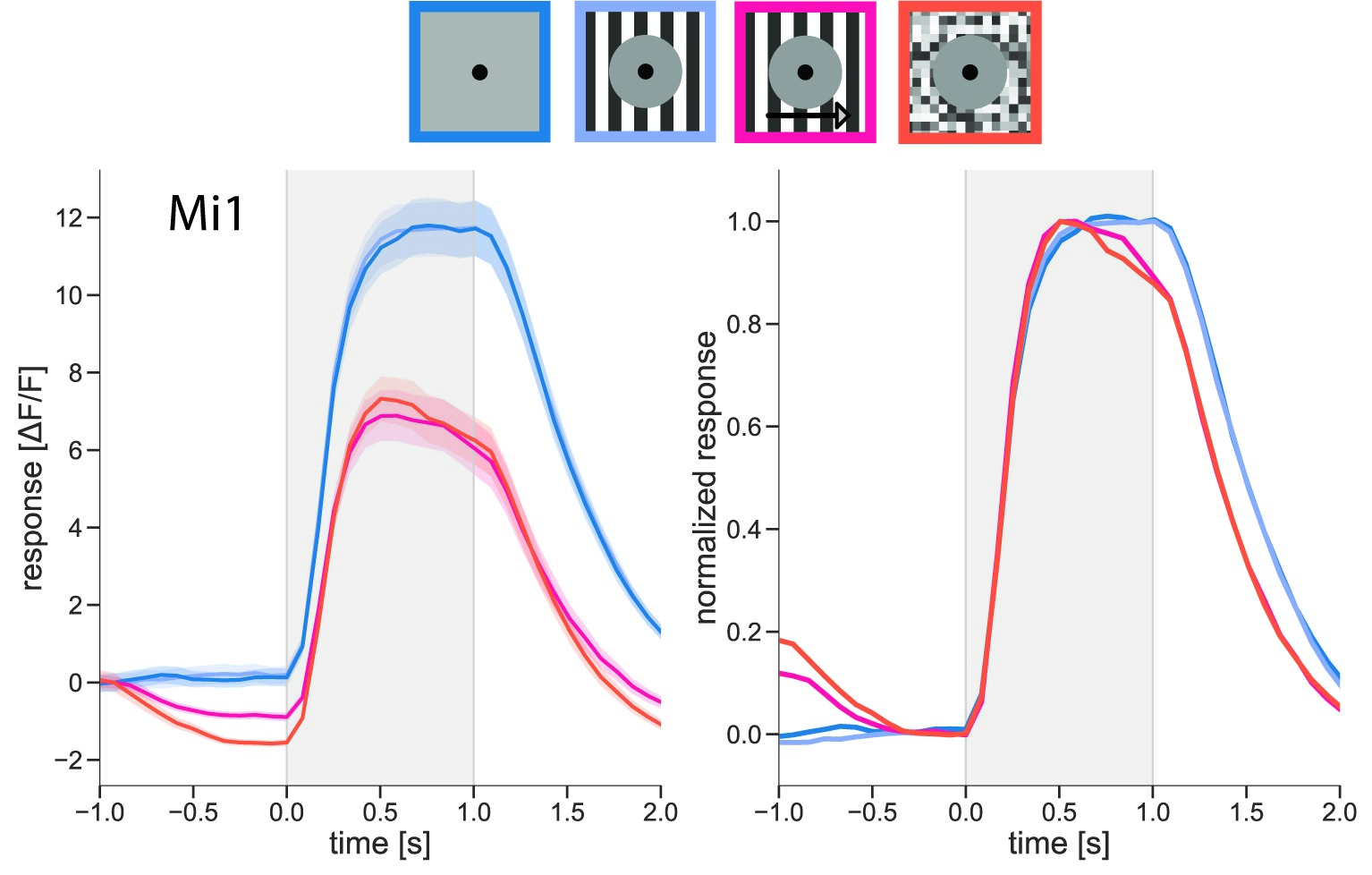

Supplement: S1 Fig — Mi1 responses to a luminance step in the RF center with gray, stationary, moving grating, and stochastic stimulus surround; n = 48 cells/11 flies. Luminance step occurred during the gray-shaded period. Left: Amplitudes of cell responses. Shaded areas around the curves show bootstrapped 68% confidence intervals. Right: Kinetics of cell responses. Responses during each condition are normalized to the condition’s maximum. (TIF) [file pone.0285686.s001.tif]

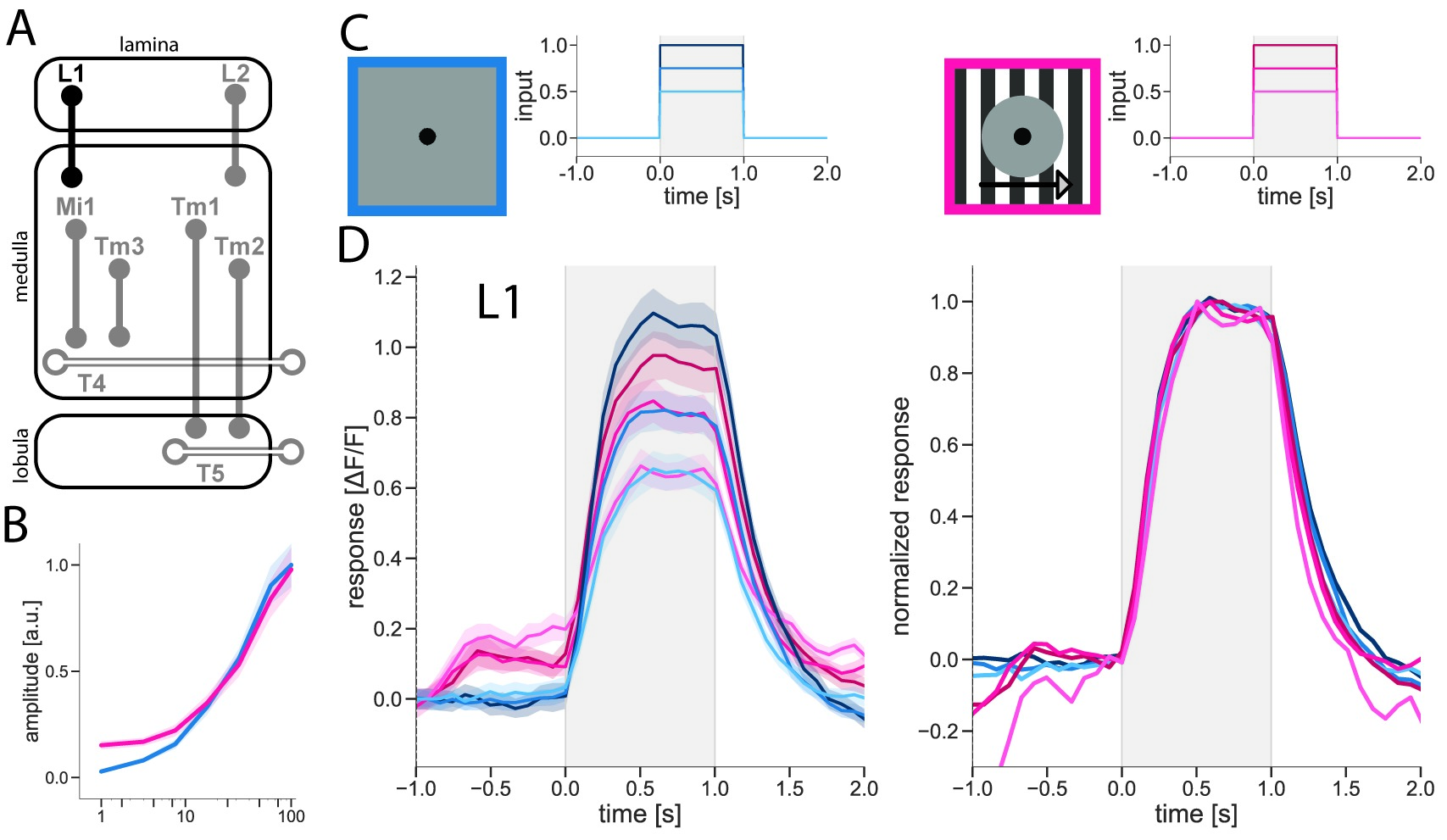

Supplement: S2 Fig — (A) Schematic representation of early stages of the motion detection circuit. Highlighted is a lamina neuron in the ON pathway that doesn’t exhibit contrast normalization properties and provides major input to contrast normalization-exhibiting neurons. (B) Contrast tuning curves for lamina neuron L1 (n = 14 cells/4 flies). Shaded areas around the curves show bootstrapped 68% confidence intervals. Adapted from Drews et al. (2020) [19]. (C) Spatial and temporal stimulus profile. Left: 3 luminance step amplitudes with gray surround. Right: 3 luminance step amplitudes with moving grating surround. (D) L1 responses to luminance steps of different amplitudes with gray and moving grating surround. Luminance step happened during gray-shaded period. Left: Amplitudes of cell responses. Shaded areas around the curves show bootstrapped 68% confidence intervals. Right: Response kinetics. Responses during each condition are normalized to condition’s maximum. (TIF) [file pone.0285686.s002.tif]

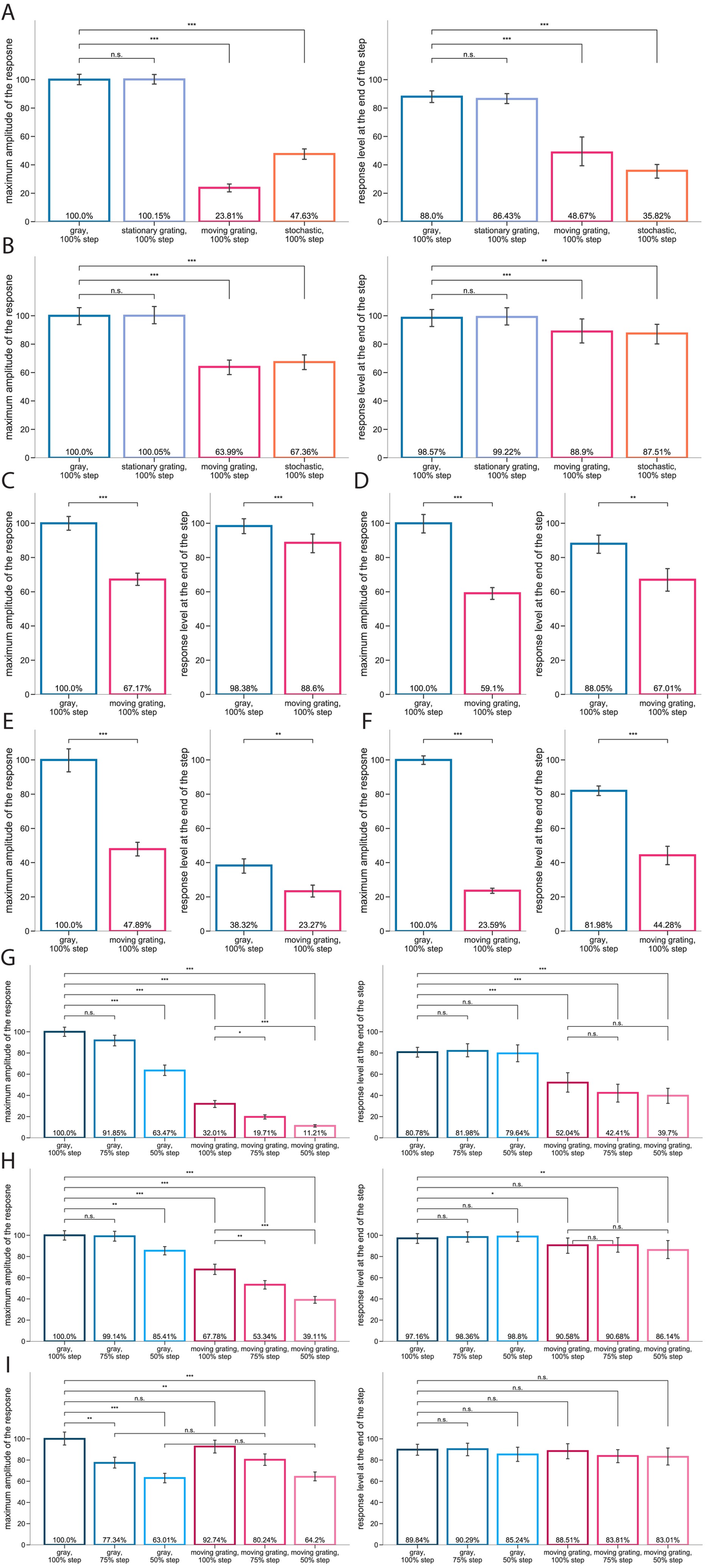

Supplement: S3 Fig — (A-I) Left: Maximum signal amplitude reached in each condition as a percentage of peak signal amplitude reached in gray surround condition. Right: Level of signal decay reached by the end of the luminance step (between 0.9 and 1.1 seconds from the start of stimulation) in each condition, as a percentage of the peak signal amplitude reached in that condition. (A) Statistics for Tm3 responses in Fig 2. Left: Amplitudes of cell responses. In comparison to the gray surround condition, response amplitude is the same in stationary grating surround condition (Mann-Whitney U: 2357, NS p = 0.46), and is suppressed when a moving grating (Mann-Whitney U: 121, ***p < 0.001) or a stochastic stimulus (Mann-Whitney U: 535, ***p < 0.001) is presented in the surround. Right: Kinetics of cell responses. In comparison to the gray surround condition, the signal decays to the same level in the stationary grating surround condition (Mann-Whitney U: 2347, NS p = 0.44), and decays significantly stronger if a moving grating (Mann-Whitney U: 777, ***p < 0.001) or a stochastic stimulus (Mann-Whitney U: 499, ***p < 0.001) is presented in the surround. (B) Statistics for Mi1 responses in S1 Fig. Left: Amplitudes of cell responses. In comparison to the gray surround condition, the amplitude of the signal is the same in stationary grating surround condition (Mann-Whitney U: 10345, NS p = 0.49), and is suppressed when a moving grating (Mann-Whitney U: 6210, ***p < 0.001) or a stochastic stimulus (Mann-Whitney U: 6223, ***p < 0.001) is presented in the surround. Right: Kinetics of cell responses. In comparison to the gray surround condition, the signal decays to the same level in the stationary grating surround condition (Mann-Whitney U: 10266, NS p = 0.44), and decays stronger if a moving grating (Mann-Whitney U: 8101, ***p < 0.001) or a stochastic stimulus (Mann-Whitney U: 8545, **p = 0.005) is presented in the surround. (C) Statistics for Mi1 responses in Fig 3. Left: Amplitudes of cell response [file pone.0285686.s003.tif]

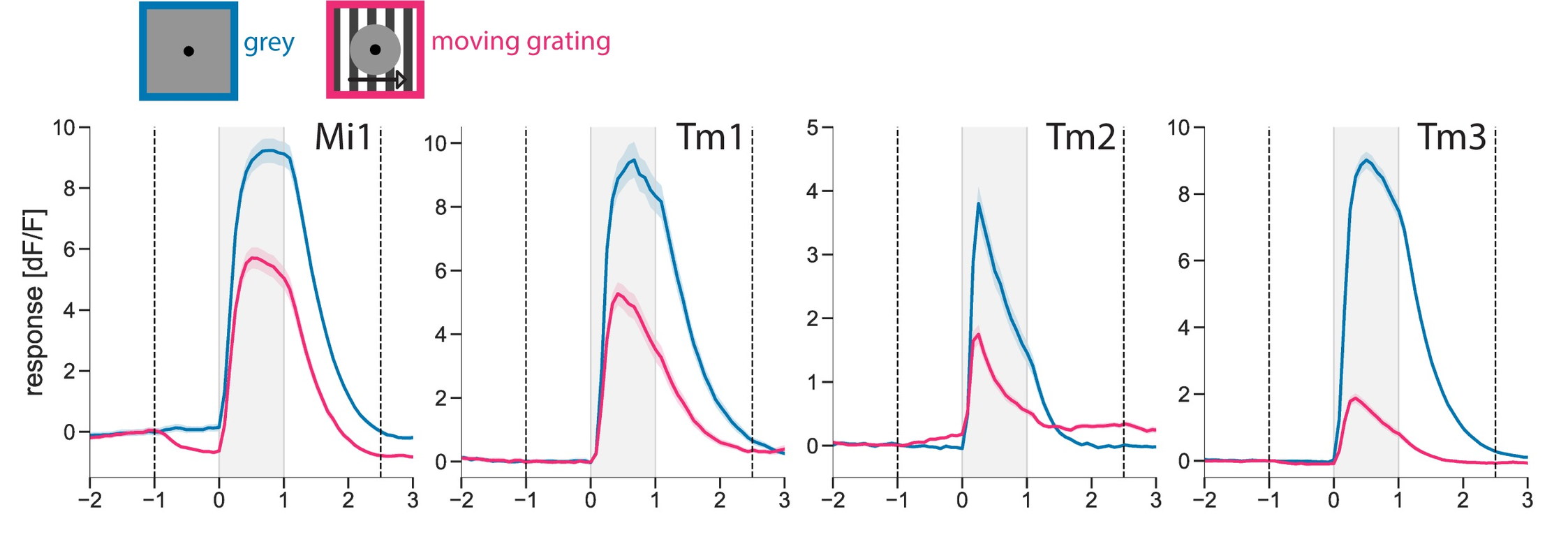

Supplement: S4 Fig — Extended traces of Fig 3 average responses of contrast normalization-exhibiting neurons to luminance step in the RF center with gray and moving grating surround. Mi1 (n = 98 cells/25 flies); Tm1 (n = 24 cells/9 flies); Tm2 (n = 22 cells/7 flies), and Tm3 (n = 65 cells/16 flies). Luminance step happened during the gray-shaded period. The dashed lines denote the time when the surround was on screen. Shaded areas around the curves show bootstrapped 68% confidence intervals. (TIF) [file pone.0285686.s004.tif]
