# Supplementary material for: Contrast normalization affects response time-course of visual interneurons
Source: PLoS One. 2023 Jun 9;18(6):e0285686. doi: 10.1371/journal.pone.0285686 (PMC10256145; doi:10.1371/journal.pone.0285686)
Supplement: Attachment — Submitted filename: Response to Reviewers.docx [file pone.0285686.s005.docx]

Dear Editor,

Thank you for considering our manuscript entitled ‘Contrast Normalization Affects Response Time-Course of Visual Interneurons’ for publication. We appreciate the reviewers' thoughtful comments and suggestions on our work. We have addressed each of the reviewers' comments below.

**Reviewer #1: Major comments**

**1) The introduction to the paper is missing previous work that has been done both in larger flies (e.g. Harris Neuron 2000) and in Drosophila (e.g. recent papers from the Silies lab). Additionally, the Matulis 2019 should be mentioned already in the introduction as part of the background for the current work.**

We added the citations to previous work [37] Harris et al. (2000) and [38] Ketkar et al. (2022) in the introduction and corrected the introduction to include Matulis et al. (2020) as part of the background.

**2) The paper is missing statistical tests. All claims regarding changes in amplitude and kinetics should be explicitly tested with the appropriate statistical test (e.g. Drews 2020 paper, Fig 5).**

We have added a figure (S3) that shows the results of Mann-Whitney U test (as in Drews et al., 2020) for each of the existing figures, included the indications of significance in the manuscript, and referred to figure S3 in the text.

**3) Authors’ choice of variability measure throughout the paper is a bit unorthodox (68% bootstrapped confidence interval). If authors choose to continue to use it, they should both justify the selection and present at least some of their results in the supplementary material with a more conventional statistics of variability.**

We use 68% bootstrapped confidence interval in the figures when the whole trace of the response is presented, as in Drews et al. (2020). We have now added figure S3 with additional statistical analysis for each of the main and supplementary figures that show 68% bootstrapped confidence interval as a measure of variability.

**4) Authors should present the entire stimulus trace, from before the surround presentation to after its disappearance. This should be done at least once for each cell type so that it will be clear to readers that the surround is indeed a classic surround that does not evoke a response.**

We have added a figure (S4) that shows extended traces of the responses from Fig. 3 for each cell type and referred to figure S4 in the text.

**Comments**

**1) State explicitly that the numbers in the figure legends are for neurons and flies.**

The legends have been corrected to explicitly state the number of cells and flies in the dataset.

**2) In Figures 2,3 and 5, the figure legend claims that responses are normalized to the cell’s maximum, but the y-axis seems to represent a non-normalized response (simple df/f).**

We apologise for the mistake. We have corrected the legends accordingly.

**3) Please clarify if the Tm3 dataset in Fig 3 contains the neurons from fig 2 or if it is completely independent.**

The Tm3 and Mi1 dataset in Fig 3 contains the data from Fig 2 and Fig 3. The legend for Fig 3 was corrected accordingly.

**4) Please address the large difference in the numbers of cells imaged for the different cell types (i.e. 98 Mi1s vs. 22 Tm2)**

Mi1 was imaged with a number of protocols that had different combinations of stimuli that all contained the ‘grey surround’ condition and the ‘moving grating’ condition presented in Fig 3. This resulted in a smaller number of Mi1 cells stimulated with a stochastic surround that was only in one stimulus set, but a higher number of responses (from all the imaged Mi1 cells) to the two conditions shown in Fig 3. These different stimulus protocols were combined into one when also other cell types were recorded, resulting in a lower number of the other cells imaged.

**5) The Matulis 2020 paper does contain local stimuli as part of their stimulus set. The explanation for the discrepancy in the discussion should be corrected.**

We apologise for the mistake. We have corrected the explanation accordingly.

**6) Included code for figure replication is using a function that has been removed since 2020 (tsplot - https://seaborn.pydata.org/whatsnew/v0.10.0.html?highlight=tsplot). Please correct to updated versions of python and associated packages.**

We have now provided a yaml file in the GitHub repository to recreate a Python environment that contains all the Python packages needed to reproduce the figures.

**7) Line 170 states that "on average, over 90% of all cells measured passed the SNR criterion". Please clarify the sentence.**

To pass the SNR criterion to become included in the data, the inter-trial variance of a cell’s responses had to be smaller than the average cell response. Large inter-trial variance was typically caused by movement artefacts. Averaged over all cell types recorded, less than 10% of the cells were discarded due to the SNR criterion.

**Reviewer #2 Minor comments/suggestions:**

**(1) Temporal dynamic is the focus of this study but was only presented in a qualitative way. It would be very helpful if the authors could quantify the effects or parameterize the temporal dynamics, and potentially compare with the modeling results.**

We parameterized and quantified the temporal dynamics of the response by measuring the level to which the response has decayed at the end of the luminance step as percent of the peak responses. We have now also added a figure (S3) that shows the results of Mann-Whitney U test (as in Drews et al., 2020) for each of the existing figures, included the indications of significance in the manuscript, and referred to figure S3 in the text.

**(2) I am not sure if the presentation of the stationary nonlinearity model is really necessary. On the other hand, must the dynamic nonlinearity model utilize the alteration of input resistance ? I suppose not. The comparison of these two very different types of models seems odd. The authors could do a better job to introduce the models.**

We still think that our attempt to explain the phenomenon with a stationary nonlinearity first makes sense, since this is the classical way to describe contrast normalization. Furthermore, even this stationary saturation nonlinearity affects the time-course of the signal due to signal compression for large amplitudes. We actually learned a lot from it: if this is the explanation, we should obtain the same signal amplitude and time course for a static surround that we observed for a moving surround by simply reducing the stimulus amplitude accordingly. Clearly, the results contradicted this explanation. On the other hand, the biophysical model could reproduce the experimental results well.

To be clearer on this, we now explicitly introduce an inhibitory input which acts as a shunt, instead of saying that the leak conductance is enlarged by the moving surround.

**(3) The receptive field sizes for the medulla neurons (described in 241-243) appear to be different from those described in Arenz et al., 2017.**

The numbers are taken from S1 and S2 tables in Arenz et. al., 2017 (Mi1 - 28.81°, Tm3 - 11.91°, Tm1 - 27.14°, Tm2 - 30.52°) and rounded to the nearest integer, resulting in the values used (29° for Mi1 cells, 12° for Tm3 cells, 27° for Tm1 cells, and 31° for Tm2 cells).

We clarified the way the values were rounded in the manuscript.
